# University students' experiences of sexual harassment: the role of gender and psychological resilience

**DOI:** 10.3389/fpsyg.2023.1202241

**Published:** 2023-07-13

**Authors:** Christina Athanasiades, Dimitrios Stamovlasis, Thanos Touloupis, Hara Charalambous

**Affiliations:** ^1^Department of Psychology, Aristotle University of Thessaloniki, Thessaloniki, Greece; ^2^Department of Philosophy and Education, Aristotle University of Thessaloniki, Thessaloniki, Greece; ^3^Department of Psychology, University of Western Macedonia, Florina, Greece; ^4^Department of Mathematics, Aristotle University of Thessaloniki, Thessaloniki, Greece

**Keywords:** sexual harassment, perceived consequences, gender, resilience, academia

## Abstract

This study aimed to investigate university students' experiences of different types of sexually harassing behaviors, within academia, as well as the role of gender and psychological resilience regarding their victimization and its consequences. Overall, 2,134 students (70.5% women), both undergraduates (81%) and postgraduates (19%), completed a self-reported online questionnaire regarding the variables involved (sexual harassment, consequences, and resilience). According to the results, the most prevailing types of sexually harassing behaviors, which were experienced mainly by women students, included offensive sexual comments/jokes/stories, inappropriate comments about one's body/appearance/sex life, as well as obscene ways of staring, obscene gestures, and/or exposure of body parts causing embarrassment. Accordingly, the perceived psycho-emotional and academic consequences of sexual harassment were more pronounced in the case of women. Furthermore, psychological resilience was negatively associated with gender, making women with low resilience more vulnerable to experiences of sexual harassment and more affected by its consequences. This study highlights important aspects of this gender-based aggressive behavior in academia and emphasizes the necessity for the implementation of appropriate policies and interventions in higher education institutions against sexual harassment.

## 1. Introduction

The phenomenon of sexual harassment in academia is a complex and multidimensional issue that concerns all members of the academic community and raises significant challenges, particularly in terms of how to address it. According to the European Directive 2002/73/EC (also called the EU Gender Directive), the term sexual harassment refers to “any form of unwanted verbal, non-verbal, or physical conduct of a sexual nature, which has the purpose or results in violating the dignity of an individual, in particular when it creates a threatening, hostile, degrading, humiliating, or offensive environment” (Hoel and Vartia, 2018, p. 13). The abovementioned behaviors may occur either in the physical space (in a variety of social contexts) and/or in cyberspace; it appears to constitute harassment both for those who directly experience them as well as for those who perceive them indirectly (as witnesses) in their environment (Johnson et al., 2018; Kasdagli and Mourtzaki, 2020).

It is well known that sexual harassment affects mostly women—especially young working women, students, and minorities—at a much higher rate than men, with serious negative implications on their overall functioning, physical and mental health, and work or academic performance (European Union Agency for Fundamental Rights, 2014; Hoel and Vartia, 2018; Swedish Research Council, 2018). Several studies have confirmed the high epidemiological incidence of sexual harassment in university and research organizations, which are characterized by precarious working conditions and hierarchical relations between employees and students, along with a culture that normalizes gender-based violence and silences the phenomenon (Johnson et al., 2018; Bondestam and Lundqvist, 2020). Rates of sexual harassment against women students in European universities vary widely, ranging, on average, from 20 to 50%, with women students in the fields of medicine and engineering suffering more than the rest of the population (Swedish Research Council, 2018; Bondestam and Lundqvist, 2020). Studies carried out in American universities also report victimization rates of up to 48% (Cantor et al., 2015).

A recent large-scale study in Europe about gender-based violence and sexual harassment in research-performing institutions, in nine European countries (https://unisafe-gbv.eu/), found that gender-based violence is relatively uniform across countries and unrelated to the work or study environment/context (Humbert et al., 2022). In addition, respondents identifying as women were more at risk of sexual violence and harassment compared to men, who were more at risk of physical violence. Finally, the same study revealed that disclosing any form of gender-based violence is systematically associated with feeling less safe, feeling unwell, and with lower work productivity or study performance, especially for women and non-binary people (Humbert et al., 2022).

Regarding consequences of sexual violence and harassment, studies mention that university students, especially women, experience a serious impact on their wellbeing as they become vulnerable to psychological distress, substance abuse problems, depression, anger, low life satisfaction, and physical illnesses (Rospenda et al., 2000; Buchanan et al., 2009; Cantor et al., 2015; McGinley et al., 2016; Wolff et al., 2017; Jirek and Saunders, 2018). Furthermore, sexually abused students report limited academic engagement, low academic achievement, as well as a generalized sense of insecurity within the university environment (Cipriano et al., 2022).

In addition to gender, another factor associated with abusive experiences is psychological resilience. Resilience is defined as the individual's ability for positive adjustment despite the existence of difficult and adverse circumstances and despite exposure to risk factors (Masten, 2001; Luthar, 2006). Resilience in early adulthood tends to be considered a crystallized psycho-emotional trait (Connor and Davidson, 2003), which is likely to act, in general, as a protective factor against risky behaviors (Scales and Leffert, 2004; Silbereisen and Lerner, 2007; Hinduja and Patchin, 2017). In other words, individuals who feel that they can successfully overcome new, unexpected, and/or difficult situations (high resilience) are considered less vulnerable and are less likely to become victims of bullying and/or harassment (Moldovan and Macarie, 2019; Thambo et al., 2019). However, since recent findings suggest that students' psychological resilience does not seem to predict their sexual harassment (Jenkins et al., 2021), more research is needed to better determine this relationship and whether resilience is a protective factor against victimization or not for women and men students.

Nevertheless, the issue of sexual harassment in academia, especially in countries and universities of South Europe, is still significantly under-investigated. It seems that more research is needed to better understand the nature and extent of the phenomenon as well as the factors that contribute to its perpetuation, which should be taken into account in the development of appropriate interventions (Grigoriou, 2010; Kambouri, 2021).

This study aimed to investigate university students' experiences of different types of sexually harassing behaviors, within academia, as well as the role of gender and psychological resilience regarding their victimization and its consequences. The study is part of a wider research project on the issue of gender-based violence in higher education institutions, conducted under the auspices of Aristotle University's Gender Equality Committee and the Center for Social Research and Decision-Making. The research took place in a large public university in a southern European country (Greece), where there is still no institutionalized provision for the prevention and response to such incidents against students and staff. More particularly, the hypotheses formulated are as follows:

Hypothesis 1: Reported experiences of different types of sexually harassing behaviors will differ based on gender and will be of a greater extent for women (Swedish Research Council, 2018; Bondestam and Lundqvist, 2020).

Hypothesis 2: Perceived consequences of sexually harassing behaviors will be worse for women students compared to men (Rospenda et al., 2000; Buchanan et al., 2009; Wolff et al., 2017; Jirek and Saunders, 2018).

Hypothesis 3: Psychological resilience will be associated both with the reported experiences of different types of sexual harassment (3a) and the perceived consequences of harassment (3b) although in a different way between genders (Moldovan and Macarie, 2019; Thambo et al., 2019).

## 2. Method

### 2.1. Procedure

After securing the study approval from the University's Research Ethics Committee (REC), data were collected from November 2021 to February 2022. The participants completed a self-reported online questionnaire anonymously. The questionnaire was uploaded on a web-based form via LimeSurvey, accompanied by a cover letter clarifying all the necessary information about the study. Without being able to locate the students' IP addresses, the link of the questionnaire was sent to all students' academic emails twice (in November 2021 and January 2022) until the necessary number of completed questionnaires was collected. As required by the study's ethics protocol, data collection followed all the principles and guidelines of the REC.

### 2.2. Sample

The sample comprised 2,134 university students (81% undergraduates). Regarding gender, 70.5% of the students identified as women, 27.0% identified as men, while 2.5% either did not identify their gender or identified as “other” in relation to gender identity. Due to the limited numerical representation, this last category of students' gender was included only in the descriptive statistics. Students' ages ranged between 18 and over 40 years old (distribution: 78.5% 18–24; 11.0% 25–29; 6.4% 29–39; and 4.1% <40). In terms of year of study, students were classified as follows: 30.3% were freshmen, 21.4% were sophomores, 16.4% were juniors, 14.7% were seniors, and 17.2% were in their fifth year or higher. Finally, students came from the following fields of study: humanities (25.3%), social sciences (20.5%), natural/physical sciences (29.8%), technological sciences (13.5%), and medical/health sciences (10.9%).

### 2.3. Instrument

The instrument of the study was an online questionnaire, which included initial demographic questions and the following two main parts:

(a) *Sexual harassment scale*. For the investigation of the students' experiences of sexually harassing behaviors and their perceived consequences, a part of a larger questionnaire, created for the same purpose by the Association of American Universities, entitled “Campus Climate Survey on Sexual Assault and Misconduct” (Cantor et al., 2020), was applied. After securing permission from the authors, the questionnaire was translated into Greek and adapted to the needs of this study. This part of the questionnaire included seven questions concerning different types of sexual harassment (see, Table 1), which were answered dichotomously (yes/no), as well as four questions regarding the perceived consequences of sexual harassment on academic achievement, academic involvement, academic environment, and physical and mental health (i.e., “to what extent the experience of harassment affected your academic achievement?” or “to what extent the experience of harassment created a hostile or offensive academic environment?”). These questions were answered on a 5-point Likert scale (from 1 = *Not at all* to 5 = *Very much*). The internal consistency coefficients Cronbach alpha for the two parts were 0.77 and 0.84, respectively.

**Table 1:** Descriptive statistics-different types of sexual harassment between genders.

**During your studies so far, has there been a time when a member of the student community or any other person working in the university…**		**Women**		**Men**		**“Other”**		**Total**	
		**N**	**%**	**N**	**%**	**N**	**%**	**N**	**%**
D1	…made offensive sexual comments or sexual jokes or told sexual stories that offended you?	272	76.40	66	18.54	18	5.06	356	22.36
D2	…made inappropriate or offensive comments about your or someone else's body, appearance, or sex life?	357	73.61	105	21.65	23	4.74	485	30.46
D3	…made rude or vulgar sexual comments to you or tried to get you to talk about sexual matters when you didn't want to?	101	71.63	30	21.28	10	7.09	141	8.86
D4	…insisted on asking you out on a date, for drinks or sex, even though you had already refused?	112	87.50	13	10.16	3	2.34	128	8.04
D5	…used the internet or social media to send or distribute sexually offensive comments/jokes/stories/photos/videos to or about you?	43	67.19	18	28.13	3	4.69	64	4.02
D6	…stared at you in an obscene way (e.g., at parts of your body) or made obscene gestures (e.g., whistling, winking) or exposed parts of his/her body, causing you embarrassment?	290	89.23	18	5.54	17	5.23	325	20.42
D7	…kissed or caressed/touched you against your will?	78	83.87	12	12.90	3	3.23	93	5.84
	**Total number of references**							**1.592**	**100.0**

(b) *Resilience scale*. Students' psychological resilience was measured through the short Greek version of the Connor-Davidson Resilience Scale (CD-RISC-10) (Connor and Davidson, 2003), following the authors' permission to use the scale. Since the multifactorial structure of the original scale (25 items) is often considered unstable, the short version was chosen whose unifactorial structure seems to have good psychometric properties (α = 0.85) in a sample of university students (Campbell-Sills and Stein, 2007). Thus, in the short version of the CD-RISC, resilience is measured through 10 representative statements/proposals (e.g., “I am able to adapt to change” or “I can handle unpleasant feelings”), which reflect individuals' ability to tolerate experiences such as change, personal problems, illness, pressure, failure, and painful feelings. The answers are given on a 5-point Likert scale (from 0 = *Not at all true* to 4 = *Almost always true*). Testing the psychometric properties of the scale, confirmatory factor analysis showed a high fit of the measurement model [χ^2^ = 126.609, *df* = 35, *p* < 0.001; CFI = 0.991; TLI = 0.988; GFI = 0.995; RMSEA = 0.032; 90% CI of RMSEA = (0.029; 0.042); SRMR = 0.037; NFI = 0.987], while reliability analysis using Cronbach's Alpha (α) and McDonald's omega (ω) also indicated high internal consistency, α = 0.836 and ω = 0.839, respectively.

## 3. Results

The statistical analyses included the following variables: gender, students' experiences of different types of sexually harassing behaviors, students' perceived consequences of sexual harassment, and resilience. Descriptive statistics, bivariate tests, such as *t*-test for independent samples, chi-square test, correlations analysis, and multivariate modeling, such as analysis of covariates (ANCOVA) and binary logistic regression, were carried out to explore the association among the variables under study.

### 3.1. Descriptive statistics and bivariate tests

Regarding the different types of sexual harassment, Table 1 shows that, among the total sample of students, the most frequent responses were (a) offensive comments about one's body, appearance, or sex life (30.46%), (b) offensive sexual comments or sexual jokes or sexual stories (22.36%), and (c) obscene staring, obscene gestures, or exposure of body parts (20.42%). The frequencies for women were in all cases higher than the corresponding frequencies for men and chi-square tests showed that the differences between genders were all statistically significant (*p* < 0.001). Among those who reported sexual harassment, rates among women (compared to men) were particularly high, exceeding 80% for the following types of harassing behaviors: (a) obscene staring, obscene gestures, or exposure of body parts, (b) unwanted insistence for a date, for drinks, or for sex, and (c) unwanted kissing or touching.

Table 2 presents the mean and standard deviation for the perceived negative consequences of sexual harassment for the two genders. The significance of the differences between the two genders was tested through *t*-tests for independent samples. According to the results, women students perceived that sexually harassing experiences had affected their academic performance, their participation in academic life, the creation of an intimidating/hostile academic environment, and their physical or mental health to a greater extent compared to men.

**Table 2:** Descriptive statistics for students' perceived consequences of sexual harassment between genders, and *T*-tests for the differences between genders.

**Perceived negative consequences**	**Gender**	***N***	**Mean**	**SD**	***T*** **-test**		
					***t***	***df***	**Sig**.
To academic performance	Women	516	1.66	0.968	−3.297	636	< 0.001
	Men	122	1.37	0.835			
To participation in academic life	Women	516	1.74	1.080	−2.685	636	< 0.01
	Men	122	1.48	0.964			
To the academic environment	Women	516	2.20	1.157	−3.915	636	< 0.001
	Men	122	1.74	1.205			
To physical/mental health	Women	516	2.14	1.162	−5.235	636	< 0.001
	Men	122	1.58	1.027			

5-point Likert scale, SD, Standard Deviation.

As far as resilience, men students (*Mean* = 3.55, *SD* = 0.656) expressed a statistically higher perceived level of resilience compared to their women peers (*Mean* = 3.32, *SD* = 0.649, *t* = 6.938, *df* = 2,077, *p* < 0.001).

In addition, a correlation analysis between perceived negative consequences of sexual harassment and resilience for both genders was performed. Specifically, the results showed that in the case of men students, there were negative correlations between resilience and all perceived consequences of harassment, that is, in relation to academic performance, participation in academic life, the academic environment, and physical/mental health (from *r* = −0.188, *p* < 0.05 to *r* = −0.344, *p* < 0.01). In the case of women students, however, there was a negative correlation only between resilience, on the one hand, and the way they perceived the academic environment as intimidating or hostile (*r* = −0.137, *p* < 0.01) as well as their physical/mental health (*r* = −0.188, *p* < 0.01), on the other hand.

### 3.2. Multivariate models

To investigate the association of students' gender with their experiences of different types of sexually harassing behaviors, considering resilience as a covariate variable, a binary logistic regression (BLR) (Agresti, 2007) was implemented. In the BLR, the log_e_[odds] of declaring an experience of a type of sexual harassment vs. the log_e_[odds] of not declaring it is modeled as a function of gender and resilience. Odds express the relative probability of the two possible responses. The results are depicted in Table 3, where the coefficients B's represent the magnitude of the effects, and the Wald statistic tests the null hypothesis (Ho) that B = 0. Larger absolute values of B denote greater effects. B's for resilience were negative in all cases, that is, the lower the resilience, the greater the relative possibility to report the particular type of sexual harassment. The interpretation is provided by Exp (B). This suggests that the estimated odds for declaring a type of sexual harassment is multiplied by the Exp (B), as resilience decreases by one unit. The findings were theoretically anticipated and interpretable.

**Table 3:** Results from a logistic regression applied to declared experiences of different types of sexual harassment, as a function of gender and resilience.

**Dependent variables (different types of sexual harassment)**	**Predictors**	**B**	**S.E**.	**Wald**	**Sig**.	**Exp(B)**	**95% C.I. for EXP(B)**	
							**Lower**	**Upper**
**D1**	*Gender*	0.480	0.148	10.474	0.001	1.616	1.209	2.162
	*Resilience*	−0.258	0.091	7.960	0.005	0.773	0.646	0.924
**D2**	*Gender*	0.279	0.125	4.965	0.026	1.322	1.034	1.689
	*Resilience*	−0.259	0.081	10.229	0.001	0.771	0.658	0.904
**D3**	*Gender*	0.228	0.216	1.115	0.291	1.256	0.823	1.919
	*Resilience*	−0.195	0.138	2.013	0.156	0.823	0.628	1.077
**D4**	*Gender*	1.195	0.299	15.992	0.000	3.302	1.839	5.931
	*Resilience*	−0.255	0.142	3.238	0.072	0.775	0.587	1.023
**D5**	*Gender*	−0.146	0.288	0.256	0.613	0.864	0.491	1.521
	*Resilience*	−0.250	0.197	1.616	0.204	0.779	0.530	1.145
**D6**	*Gender*	1.976	0.249	62.931	0.000	7.211	4.426	11.748
	*Resilience*	−0.130	0.097	1.787	0.181	0.878	0.726	1.062
**D7**	*Gender*	0.849	0.316	7.217	0.007	2.337	1.258	4.342
	*Resilience*	−0.461	0.164	7.910	0.005	0.630	0.457	0.869

The regression coefficient B, standard deviation (B), Wald Test value, significance (sig), Exp(B) and the 95% confidence intervals of Exp(B). For gender, the group of comparison is men.

Regarding gender, the group of comparison was men, and B represents the increase in effect with respect to women. For example, in the first type of sexual harassment (D1) for women, the effect increases by 0.480 compared to men, and for women, the estimated odds of this type of sexual harassment are increased, multiplied by 1.616. Note that interaction terms between gender and resilience were not statistically significant.

To investigate the association between students' gender and their perceived consequences of sexual harassment, an analysis of variance with resilience as covariate (ANCOVA) was implemented. The results are shown in Table 4, wherein the coefficients Bs represent the magnitude of the effects and the *T*-tests account for rejecting the null hypothesis (Ho) that B = 0. Larger absolute values of B denote greater effects. All B's for resilience were negative, that is, the lower the resilience, the greater the perceived consequences of sexual harassment.

**Table 4:** Results of ANCOVA of students' perceived consequences of sexual harassment, with gender as independent variable and resilience as covariate.

**Dependent variables (perceived negative consequences of sexual harassment)**	**Independent variables**	**B**	**Std. Error**	***t***	**Sig**.	**95% Confidence Interval**		**Partial *n*^2^**	**Observed Power**
						**Lower**	**Upper**		
To academic performance	Gender (Women)	0.257	0.095	2.69	0.007	0.070	0.444	0.011	0.767
	Gender (Men)	0^a^							
	Resilience	−0.136	0.055	−2.48	0.013	−0.244	−0.029	0.010	0.699
To participation in academic life	Gender (Women)	0.234	0.107	2.19	0.029	0.024	0.444	0.007	0.588
	Gender (Men)	0^a^							
	Resilience	−0.153	0.062	−2.49	0.013	−0.274	−0.032	0.010	0.700
To the academic environment	Gender (Women)	0.400	0.117	3.42	0.001	0.171	0.630	0.018	0.928
	Gender (Men)	0^a^							
	Resilience	−0.279	0.067	−4.14	0.000	−0.411	−0.147	0.026	0.985
To physical/mental health	Gender (Women)	0.478	0.113	4.24	0.000	0.256	0.699	0.027	0.988
	Gender (Men)	0^a^							
	Resilience	−0.364	0.065	−5.60	0.000	−0.491	−0.236	0.047	1.000

^a^Group of comparison.

Regarding gender, the group of comparison was men, and B represents the increase in effect with respect to women. For example, in the perceived consequence of reduced academic performance, an increase of 0.257 is observed for women students compared with men students. Note that interaction terms between gender and resilience were not statistically significant.

## 4. Discussion

The present study aimed to investigate students' experiences of different types of sexually harassing behaviors, within academia, as well as the role of gender and psychological resilience regarding their harassment and its consequences.

According to the university students' self-reports, the most prevailing types of sexually harassing behaviors in academia included offensive sexual comments, jokes, or stories, inappropriate or offensive comments about one's body, appearance, or sex life, as well as the obscene way of staring (e.g., at parts of your body), the obscene gestures (e.g., whistling and winking), or the exposure of one's body parts that causes embarrassment. In all cases, types of sexually harassing behaviors were primarily manifested against women students, with reporting rates exceeding 70% (among those students who reported incidents of sexual harassment). This finding supports Hypothesis 1 as well as recent related studies that reveal mainly women's sexual victimization in academia (Swedish Research Council, 2018; Bondestam and Lundqvist, 2020; Humbert et al., 2022). These percentages may be attributed both to the absence of long-term measures to prevent and address sexual harassment within the academic context, as well as to a broader social culture that encourages sexist attitudes and silences the phenomenon (Alldred and Phipps, 2018; Kambouri, 2021). It should be emphasized that the abovementioned harassing behaviors are examples of gender-based violence, as they affect women disproportionately compared to men, constituting a mechanism of sexist discrimination against women (Vaiou et al., 2021; Humbert et al., 2022).

Accordingly, the study showed that the perceived consequences of the different types of sexual harassment seemed to be significantly more pronounced in the case of women students. This finding supports Hypothesis 2 and is in parallel with related international studies (Rospenda et al., 2000; Cantor et al., 2015; McGinley et al., 2016; Wolff et al., 2017; Jirek and Saunders, 2018; Cipriano et al., 2022). More particularly, the effects of the phenomenon seem to impact primarily the women students' levels of physical and mental health, their academic performance, as well as their involvement in academic life, creating an overall intimidating and offensive academic environment.

Furthermore, the findings showed that the students' perceived level of resilience was associated negatively with their reported experiences of sexual harassment. To the extent that women students reported lower levels of resilience than men, as well as more experiences of sexual harassment, this may suggest a vulnerability toward victimization, as has been reported elsewhere (Touloupis and Athanasiades, 2022). However, from the analysis of covariates, it was shown that independent of the resilience effect, being a woman was associated with further reported experiences of sexually harassing behaviors.

It is worth mentioning that the negative association between resilience and harassment against women was supported not for all but only for specific types of sexual harassment, such as offensive sexual comments/jokes/stories, inappropriate/offensive comments about one's body/appearance/sex life, and being kissed or caressed/touched against one's will (see D1, D2, and D7 in Tables 1, 4). Therefore, the above finding confirms partially Hypothesis 3a, as it was expected that the perceived level of resilience will be negatively related to all types of sexual harassment against women. This is likely since the above types of sexual harassment (e.g., offensive sexual comments or jokes, inappropriate or offensive comments about one's body or appearance, and being touched unwillingly) are among the most common in the academic environment of the participants. Undoubtedly, due to the scarcity of related findings regarding all these different types of sexually harassing behaviors included in this study, future studies need to further clarify whether the negative interaction between resilience and sexual harassment against women concerns other types of harassing behaviors as well. Overall, the above findings reflect similar studies, which have reported that resilience is acting as a protective filter against sexual harassment (Moldovan and Macarie, 2019; Thambo et al., 2019). However, as previously mentioned, this study adds empirical evidence that the female gender *per se* is a predictive factor for experiencing sexual harassment.

Similarly, it was found that the students' perceived level of resilience seemed to be negatively associated with their perceived consequences of sexual harassment, while an effect of gender is present. Women perceived negative consequences of sexual harassment, especially in their academic environment as well as in their physical and mental health, were more intense compared to men. This finding supports Hypothesis 3b. Resilience, which theoretically has the potential to buffer the effect of students' experience of sexual harassment on their psycho-emotional state and their involvement in academic life and duties (Moldovan and Macarie, 2019; Thambo et al., 2019), appeared lower for women, and this could partially explain why women reported more intense consequences of sexual harassment than men. Nevertheless, independent of resilience, female gender *per se* is a predictive factor for high perceived consequences of sexual harassment.

Undoubtedly, the above findings should be interpreted cautiously due to specific limitations. More particularly, results were based on a convenient sample, while the rates of different types of sexual harassment reflect the students' responses and not the actual cases of harassment. The conduction of the study immediately after a long period of social isolation and confinement, due to the restrictive measures of the COVID-19 pandemic, may have influenced the participants' responses, regarding both sexual harassment and resilience. For example, because of distance education, a portion of the sample had not been able to fully experience student life within the university campus. In addition, further research could focus exclusively on the student community or on specific minority groups of students (i.e., women students from specific faculties, LGBTQ+ students, and students with special educational needs and disabilities), using a qualitative methodology, which would enhance the above findings, highlighting other qualitative parameters of this issue. Furthermore, besides gender and resilience, other risk factors of sexual victimization, such as stress, anxiety, depression, and sense of belonging, could be explored.

Nevertheless, the results shed light on important aspects of the phenomenon, providing a baseline for the implementation of appropriate policies and interventions in academic institutions against sexual violence and harassment. Measures that are specifically targeted to the student population (as well as to all members of the academic community) to overturn a culture of tolerance toward gender-based violence and harassment are considered important. Such measures include, for example, training seminars or information and awareness-raising campaigns for women and men on gender relations, gender equality, and acceptance of diversity. At the same time, the actions could aim at enhancing students' resilience as well as their level of wellbeing and connectedness to the wider academic community. In accordance with the relevant literature (Johnson et al., 2018; Bondestam and Lundqvist, 2020), it is recommended that academic institutions organize the above initiatives at the following levels: (a) at the policy level, by adopting firm and transparent measures against gender-based violence; (b) at the level of awareness-raising and sensitization of the academic community, aiming at an inclusive environment that honors and respects equality and diversity; (c) at the level of managing individual cases, through the establishment of relevant bodies and procedures; and (d) at the level of supporting mechanisms for the victims, independently of formal complaints.

## Data availability statement

The raw data supporting the conclusions of this article will be made available by the authors, without undue reservation.

## Ethics statement

The studies involving human participants were reviewed and approved by Research Ethics Committee of the Aristotle University of Thessaloniki (protocol number 148172/2021). The patients/participants provided their written informed consent to participate in this study.

## Author contributions

CA and HC: conceptualization, investigation, resources, and funding acquisition. CA, TT, and DS: methodology, validation, data curation, writing—original draft preparation, writing—review and editing, and visualization. DS: software. CA and DS: formal analysis. CA: supervision and project administration. All authors have read and agreed to the published version of the manuscript.
